# Comparison of outcomes of four different treatment modalities for diabetic vitreous haemorrhage

**DOI:** 10.1038/s41598-020-60378-8

**Published:** 2020-02-28

**Authors:** Ibrahim Taskintuna, Maram E. A. Abdalla Elsayed, Kaan Taskintuna, Khabir Ahmad, Rajiv Khandekar, Patrik Schatz, Igor Kozak

**Affiliations:** 1 Vitreoretinal Division, King Khaled Eye Specialist Hospital, Riyadh, Kingdom of Saudi Arabia; 2 Jeddah Eye Hospital, Jeddah, Kingdom of Saudi Arabia; 3 Research Department, King Khaled Eye Specialist Hospital, Riyadh, Kingdom of Saudi Arabia; 4 Department of Clinical Sciences, Ophthalmology, Lund University, Lund, Sweden; 5 Moorfield Eye Hospital Centre, Abu Dhabi, United Arab Emirates

**Keywords:** Medical research, Outcomes research

## Abstract

We compared outcomes of four different management modalities for diabetic VH. Patients with diabetic VH were identified in this retrospective study undertaken at King Khaled Eye Specialist Hospital, Riyadh, Saudi Arabia. Eyes were grouped based on the treatment received: control (observation only), intravitreal bevacizumab (IVB) injection(s), pars plana vitrectomy (PPV), and preoperative single IVB injection before PPV. Best-corrected visual acuity (BCVA) and status of VH were noted at baseline and the last follow up (Minimum: 6 months, maximum: 29 months). The proportion of eyes with Snellen BCVA improvement by two lines or more and VH clearance at the last follow up were compared between groups. The four groups – Control, IVB, PPV, and IVB-before-PPV had 23, 29, 17, and 20 eyes, respectively. The proportion of eyes gaining ≥2 lines was substantially higher in the IVB-before-PPV and PPV groups (90% and 77%, respectively) compared with IVB and observation groups (41% and 22%, respectively). Surgical treatment was associated with a 2.38 times higher likelihood of gaining ≥2 lines than the non-surgical one (incidence ratio: 2.38, 95% CI 1.19, 4.78 *P* = 0.015) after adjusting for age, hyperglycemia and BCVA at presentation. Less invasive treatment such as IVB injections did not result in the same amount of improvement in vision as did PPV. Prospective randomized studies are needed to better define the role of IVB injections in the management of diabetic VH.

## Introduction

Diabetic Retinopathy (DR) is a leading cause of visual impairment worldwide. The two most frequent vision-threatening complications of DR are diabetic macular edema (DME), and vitreous hemorrhage (VH) secondary to proliferative diabetic retinopathy (PDR)^1,2^. Panretinal photocoagulation (PRP) has traditionally been the standard treatment for PDR^3^. While the results of clinical trials using anti-angiogenic agents in PDR are documented, its introduction in clinical practice for diabetic vitreous haemorrhage needs further evidence^4,5^. Unfortunately, even with appropriate PRP treatment, 5% of high-risk PDR cases may require pars plana vitrectomy (PPV) for PDR related vitreous haemorrhage^6^.

Eyes with non-clearing diabetic VH are either monitored, with addition of PRP when possible, until spontaneous resolution or such eyes are treated surgically by PPV. The goal of PPV is to clear the VH and apply PRP (endolaser) during the procedure in order to induce regression of neovascularization and thereby preventing recurrent bleeding. Despite recent advances in vitrectomy techniques, there is still a risk of surgical complications related to PPV including retinal detachment, endophthalmitis, neovascular glaucoma, and phthisis bulbi^7^. Intravitreal anti-vascular endothelial growth factor (anti-VEGF) pharmacotherapy has also been found useful to reduce the complications of PDR^5^.

To date, there are only few published studies on the treatment of diabetic VH with intravitreal antiangiogenic injections. Published studies show the benefit of intravitreal bevacizumab or ranibizumab injections in VH due to diabetic retinopathy in selected cases^8–11^. Intravitreal bevacizumab (IVB) injections supress the VEGF production thus may prevent re-bleeding in the proliferative diabetic eye. The accompanying volume dilution of the VH may lead to clearance of vitreous haze and inferior displacement of the VH, thus may hasten the resolution of the VH^8^.

Spaide *et al*. reported successful treatment of VH due to PDR with IVB in 2 patients^9^. Parikh *et al*. recently reported on the role of IVB injections in the management of patients with VH secondary to PDR. In their study, they concluded most patients may be managed non-surgically^10^. Another recent study by Chelala *et al*. demonstrated intravitreal ranibizumab injections were effective in mild-moderate VH in diabetic patients^11^.

These previous studies showed promising results regarding intravitreal anti-VEGF injections in the treatment of diabetic VH, but did not compare intravitreal injections to observation and pars plana vitrectomy for the treatment of diabetic VH. We conducted this study to analyse the visual outcomes and VH clearance rates following 4 different modes of management for diabetic VH: observation, IVB injections, pars plana vitrectomy (PPV), and IVB injection-before-PPV.

## Methods

This retrospective cohort study was carried out in a tertiary care eye hospital of central Saudi Arabia between January 2015 and December 2016. This study adhered to the tenets of the Declaration of Helsinki and was approved by the Institutional Review Board of the King Khaled Eye Specialist Hospital (KKESH). Study participants were identified through search of medical records based on diagnostic coding, and their data were collected through chart review. Informed consent was obtained from the subjects for each of the procedure.

Patients with either Type 1 or 2 diabetes mellitus, over 18 years of age, presenting with VH due to PDR, were eligible for inclusion in the study. If there was bilateral VH, the eye treated first was included in the study. Patients that received IVB intravitreal injections or PPV in the past or diagnosed to have tractional retinal detachment (TRD) were excluded. TRD was ruled out by B-scan if there was no view to fundus. Patients were also excluded if VH was due to any disease other than PDR, or the follow-up was less than 6 months after presentation/treatment of VH.

Based on type of management, eyes were grouped into: control (observation only), intravitreal bevacizumab (IVB) injection(s), pars plana vitrectomy (PPV), and preoperative single intravitreal bevacizumab (IVB) injection before PPV. Eyes in the observation group were those that were observed at least 6 months for non-clearing VH without any intervention. Eyes in the PPV groups had their procedure within 1 month of their presentations. In the preoperative IVB-before-PPV group, eyes received IVB injections 48–72 hours before the PPV surgeries. In IVB group, there was no limit on the number of injections one can receive as the goal was to clear the vitreous or maintain the vision.

Demographic information, diabetes type, hypertension, hyperlipidemia, and prior PRP were recorded at presentation. The BCVA (measured using Snellen chart) and intra ocular pressure (IOP) were recorded at baseline and at each follow-up. A complete slit-lamp examination of the eyes were done at each visit. If any intervention was planned, such as either IVB injection or PPV, for group I (observation) or PPV for the IVB group, we recorded the BCVA just before the intervention as the final VA. We converted the Snellen’s chart notation of distance visual acuity into LogMAR values for the purpose of statistical analysis^12^.

The primary outcome was to compare the proportion of eyes gaining at least two lines in the best-corrected Snellen VA at last follow-up compared to baseline, across groups. The secondary outcomes were (1) mean LogMAR BCVA gain, (2) the proportion of eyes that had complete/partial clearance of VH in the visual axis at last follow-up, (3) proportion of recurrent VH, and (4) rate of additional treatment by PPV or IVB as adjuvant therapy to manage non-clearing VH in the observation and IVB injection groups after a minimum of 6 months of follow-up.

All statistical analyses were performed using IBM Statistical Package for Social science (SPSS) for Windows, version 26 (IBM, NY, USA) and GraphPad Prism version 8 (GraphPad Software, Inc.). Proportions were compared using Chi-squared test or Exact test, as appropriate. Means were compared using ANOVA. Tukey’s multiple comparison test was used to determine which means differed from the rest. Using cox regression analysis, incidence ratios with 95% CIs were calculated to determine the association between BCVA gain and the treatment. For all the analyses, 2-sided p-values were calculated. A p-value < 0.05 was considered significant.

## Results

Our cohort included 89 eyes of 89 patients with diabetic VH. At presentation, the four groups – Control (n = 23), IVB (n = 29), PPV (n = 17), and preoperative IVB-before-PPV(n = 20) – were comparable with respect to gender, type of diabetes, DME, and history of nephropathy, hypertension and PRP, but were not similar with respect to age (P = 0.0495), hyperglycemia (P = 0.006) and BCVA (P < 0.001) and lens status (P < 0.007) (Table 1).

**Table 1:** Characteristics of patients with diabetic VH receiving different treatments (n = 89 eyes).

		Treatment*								P^‡^
		Control^†^ (n = 23)		IVB (n = 29)		PPV (n = 17)		preoperative IVB-before-PPV (n = 20)		
		Freq	%	Freq	%	Freq	%	Freq	%	
Age		61.13 ± 12.46		57.40 ± 12.52		56.16 ± 13.49		49.96 ± 13.73		0.0495
Sex	Male	16	69.6	17	58.6	9	52.9	16	80	0.284
	Female	7	30.4	12	41.4	8	47.1	4	20	
Eye involved	Right	11	47.8	18	62.1	9	52.9	11	55	0.779
	Left	12	52.2	11	37.9	8	47.1	9	45	
Diabetes	Type 1	2	8.7	4	13.8	2	11.8	5	25	0.506
	Type 2	21	91.3	25	86.2	15	88.2	15	75	
Nephropathy	Yes	6	26.1	7	24.1	5	29.4	7	35	0.860
	No	17	73.9	22	75.9	12	70.6	13	65	
Hyperglycemia	Yes	13	56.5	26	89.7	15	88.2	18	90	0.006
	No	10	43.5	3	10.3	2	11.8	2	10	
Hypertension	Yes	6	26.1	9	31.0	4	23.5	8	40	0.692
	No	17	73.9	20	69.0	13	76.5	12	60	
Past PRP	Yes	16	72.7	24	82.8	11	68.8	17	85	0.567
	No	6	27.3	5	17.2	5	31.3	3	15	
Lens status	No cataract	1	4.3	2	6.9	7	41.2	5	25	0.007
	Cataract	10	43.5	19	65.5	6	35.3	11	55	
	Pseudophakia	12	52.2	8	27.6	4	23.5	4	20	
Diabetic macular edema	Yes	11	47.8	20	69.0	12	70.6	10	50.0	0.262
	No	12	52.2	9	31.0	5	29.4	10	50.0	
LogMAR BCVA at presentation		0.79 ± 0.54		0.98 ± 0.55		1.55 ± 0.32		1.33 ± 0.43		<0.001

*2 eyes in Control group and 1 eye each in IVB and PPV groups had VMT. 1 eye in PPV group had BRVO. Cornea was clear in all the study eyes.

^†^Observation only.

^‡^Means were compared using ANOVA. Proportions were compared using Chi-squared test or Exact test, as appropriate.

Overall, surgically treated eyes had worse visual acuity and belonged to younger and more hyperglycaemic individuals than the control eyes.

The mean duration of follow-up (minimum: 6 months, maximum: 29 months) was not significantly different across groups (15.09 ± 6.24, 15.48 ± 6.18, 17.76 ± 6.80, 17.76 ± 6.80 months, respectively, P = 0.137).

The proportion of eyes gaining ≥2 lines was the highest in the preoperative IVB-before-PPV group followed by PPV. Only one out of every 5 eyes in the control group gained ≥2 lines. A cox regression analysis also revealed a much higher likelihood of BCVA gain in eyes that underwent PPV (irrespective of whether or not they received an preoperative IVB injection before PPV) than those that did not receive any treatment (Table 2). For PPV and preoperative IVB-before-PPV groups compared with control, the age, hyperglycemia and BCVA adjusted incidence ratios for VA gain of ≥2 lines were 3.31(95% CI, 1.03, 10.62; P = 0.044) and 3.79(95% CI, 1.26, 11.43; P = 0.018), respectively. Overall, surgical treatment was associated with a 2.38 times higher likelihood of gaining ≥2 lines than the non-surgical one (incidence ratio: 2.38, 95% CI 1.19, 4.78 P = 0.015) after adjusting for age, hyperglycemia and BCVA at presentation.

**Table 2:** Association between treatment and BCVA gain of ≥2 lines from bassline.

Treatment	No. of treated eyes	No. (%) of eyes gaining ≥2 lines	Model 1* Crude incidence ratio (95.0% CI)	P	Model 2* Adjusted incidence ratio (95.0% CI)	P	Model 3* Adjusted incidence ratio (95.0% CI)	P
Control^†^	23	5 (21.7)	1.0		1.0		1.0	
IVB^‡^	29	12 (41.4)	1.90 (0.67, 5.40)	0.227	1.79 (0.61, 5.28)	0.289	1.79 (0.61, 5.30)	0.290
PPV	17	13 (76.5)	3.52 (1.25, 9.87)	0.017	3.30 (1.13, 9.61)	0.029	3.31(1.03, 10.62)	0.044
preoperative IVB-before-PPV	20	18 (90)	4.14 (1.54, 11.15)	0.005	3.78 (1.32, 10.83)	0.013	3.79(1.26, 11.43)	0.018

*Global p values for Model 1(Crude), Model 2(adjusted for age and hyperglycemia) and Model 3 (adjusted for age, hyperglycemia and BCVA at presentation) were 0.015, 0.035 and 0.071, respectively.

^†^Observation.

^‡^The mean number of injections was 2.66 ± 1.70 (Median(IQR):2(3), Min: 1, Max: 7).

**Figure 1:**
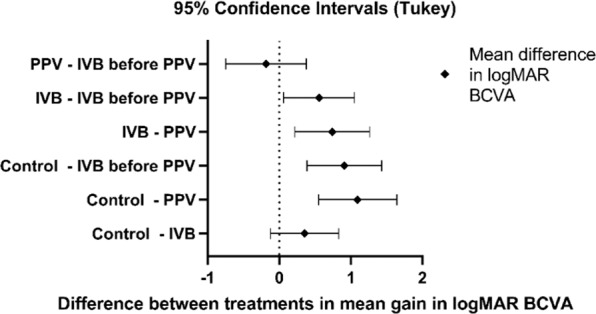
Difference between treatment groups in mean gain in logMAR BCVA.

A further analysis was done to compare the mean gain in logMAR BCVA across groups (Table 3 and Fig. 1). There was a statistically significant difference between groups as determined by one-way ANOVA (F (3.85) = 12.26, P < 0.001). A Tukey post-hoc analysis revealed that, as compared with the control group, the mean gain (a negative sign indicating gain) was significantly greater in the PPV (−1.09, 95% CI −1.64, −2.19; P < 0.001) and preoperative IVB-before-PPV (−0.91, 95% CI −1.43, −0.39, P < 0.001) groups. A similarly significant but slightly less pronounced effect was also noted when the mean gain in the PPV and preoperative IVB-before-PPV groups was compared with the IVB group (P = 0.002 and P = 0.022, respectively). No significant difference was observed between the two PPV groups (P = 0.824) and between the IVB and Control groups (P = 0.219).

**Table 3:** Comparison of mean gain in logMar BCVA between different treatment groups using Tukey’s multiple comparisons analysis (n = 89).

Treatment group	Mean gain in BCVA	95% CI of gain	Adjusted P
PPV vs. Control	−1.09	−1.64, −2.19	<0.001
preoperative IVB-before-PPV vs. Control	−0.91	−1.43, −0.39	<0.001
PPV vs. IVB	−0.74	−1.26, −0.22	0.002
preoperative IVB-before-PPV vs. IVB	−0.56	−1.05, −0.06	0.022
IVB vs. Control	−0.35	−0.83, 0.12	0.219
preoperative IVB-before-PPV vs. PPV	0.19	−0.38, 0.75	0.824

**Figure 2:**
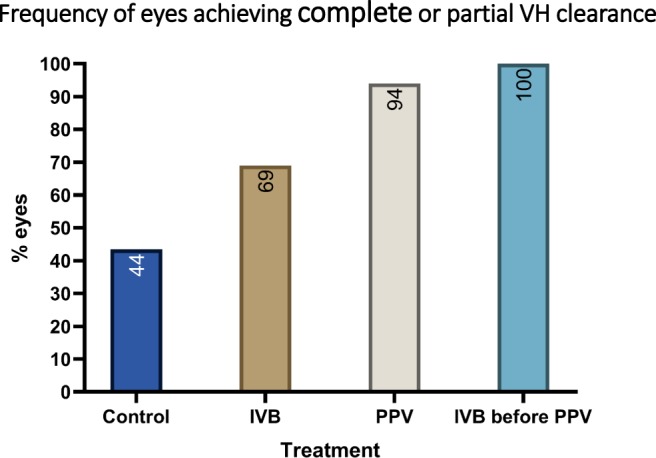
Status of vitreous hemorrhage (VH) in the treatment groups vs control at the last follow up (*P* < 0.001).

There was a statistically significant difference in the frequency of complete or partial VH clearance across groups (P < 0.001). All eyes in preoperative IVB-before-PPV group and 94.1% eyes in PPV group achieved complete or partial VH clearance, compared with only 43.5% and 69% in Control and IVB groups, respectively (Fig. 2). Non-clearing vitreous haemorrhage was main cause of poor visual improvement. 18 eyes in IVB group, 17 eyes in the IVB group, and 4 eyes in the PPV group did not gain 2 lines or more. Of these, non-clearing VH was responsible in 11, 8, 1 eyes, respectively.

One eye (4.3%) in the control group and 11 (37.9%) eyes in the IVB group developed recurrent haemorrhage during the follow-up period. In the control group, 4 (17.4%) eyes underwent PPV and another 6 (20.7%) eyes received IVB for non-clearing VH. In the IVB group, 6 (20.7%) eyes underwent PPV during the follow up. In the PPV and preoperative IVB-before-PPV groups, eight (47.1%) and 7 (35%) eyes, respectively, developed post-op VH. One eye each in the PPV and preoperative IVB-before-PPV groups underwent another PPV. In the follow-up period, three and 5 eyes in the PPV and preoperative IVB-before-PPV groups, respectively, received IVB injections for post-op VH.

None of the eyes developed retinal detachment, glaucoma or endophthalmitis during the follow up period. In the control group, of 10 eyes had some form of cataract at baseline and one eye with clear lens, none had surgery during the follow up. In IVB group, of 19 eyes with cataract at baseline, 7 had cataract surgery, of 2 eyes with clear lens at baseline, none had surgery during the follow up. In PPV group, 6 eyes had cataract at baseline. Of these, 1 eye had combined phaco + IOL + PPV surgery and 2 eyes had cataract surgery during the follow up. Of 7 with clear lens at baseline, 1 had surgery during the follow up. In preoperative IVB-before-PPV group, of 11 eyes with any form of cataract at baseline, 6 eyes had combined phaco + IOL + PPV surgery. Of 5 eyes with clear lens at baseline, none had surgery during the follow up.

## Discussion

We found evidence of beneficial effect of PPV in the treatment of diabetic VH regardless of whether it was preceded by an IVB injection. Eyes undergoing PPV (with or without a prior IVB injection) were substantially more likely to gain 2 lines or more of best corrected vision compared with the observation group or IVB injections only group.

Our study was triggered by a lack of standard of care for the treatment of diabetic VH. Observational studies have yielded poor visual outcomes^13^. A Royal College of Ophthalmologists’ National Ophthalmology Database study of 939 eyes (834 patients) undergoing primary vitrectomy for VH due to PDR in the United Kingdom showed a 13.1% intraoperative complication rate in vitrectomies without delamination of membranes. In the same study, 11.7% of the eyes required further vitrectomy due to, among other factors, recurrent VH^14^. Thus, alternative management of diabetic VH, such as potentially less invasive and safer treatment with intravitreal antiangiogenic agents, may have a role^9–11^.

Herein we presented data from a retrospective study with no strict protocol for intravitreal injections versus surgery wherein IVB injections were given at the discretion of the treating physician or driven by patient preference until the clearance of VH. It is possible that denser VH were treated with PPV rather than IVB. Despite limitations inherent to its retrospective design, this is the first study to have compared the 4 contemporary management modalities for diabetic VH. In our study, diabetes related risk factors were similar across groups apart from hyperglycemia. Hyperglycemia was more common in the treatment groups than the control group. The effect of hyperglycemia on the visual results may be minimal because there was no difference in other parameters such as nephropathy indicating disease severity across groups. Further, patients in the observation and IVB injections only groups were significantly older than those PPV groups. It is possible that some patients who were considered not fit for anaesthesia or refusing surgery received these treatments.

In our study, a substantially more vision gain in PPV groups compared to observation or IVB injections only groups is noteworthy. Baseline BCVA was significantly poorer in the PPV groups, which may indicate a tendency for dense VH being treated with PPV. One might argue that a higher mean gain occurred in the surgical groups since there was a more room for improvement than the non-surgical groups. However, there was also a higher likelihood of vision gain of 2 lines or more in the vitrectomy groups. In the Royal College of Ophthalmologists’ National Ophthalmology Database study of vitrectomy for VH due to PDR, 63.6% eyes gained 2 Snellen’s lines or more after the procedure^13^. The use of preoperative IVB was not analysed in that study. Our findings are in contrast to those by Parikh *et al*. who found no significant difference in final VA between eyes with VH that underwent PPV and those that received injections only^9^.

In our study, there was no statistically significant difference in vision gain between eyes that received or did not receive IVB as an adjunct to PPV, but the small sample size prevents us from making any firm conclusions. The value of IVB as an adjunct to PPV needs to be evaluated in more controlled studies. The intraoperative effect of the IVB that this study could not assess also need to be evaluated.

Rate of prior PRP at presentation did not significantly differ across the groups. We cannot rule out the possibility that the results would be different for the observation and IVB groups if the patients were PRP naive at presentation^9^.

In conclusion, IVB injection(s) alone did not result in the same amount of vision improvement as did PPV in the management of diabetic VH. A preoperative IVB injection may reduce the risk of recurrent VH among eyes with VH undergoing PPV. Prospective research is warranted to define the role of IVB, either as monotherapy or as an adjunct to PPV, in the management of diabetic VH.
